# Precarious employment and its associations with perceived stress and self-rated health: a Danish cross-sectional study

**DOI:** 10.1007/s00420-026-02213-7

**Published:** 2026-05-20

**Authors:** Per Høgh Poulsen, Jesper Medom Vestergaard, Trine Nøhr Winding, Karin Biering, Johan Hviid Andersen, Rasmus Juul Møberg

**Affiliations:** 1 Department of Occupational and Environmental Medicine, University Research Clinic, Danish Ramazzini Centre, Gødstrup Hospital, Herning, Denmark; 2 Department of Clinical Medicine, Faculty of Health, Aarhus University, Aarhus, Denmark; 3 Department of Occupational and Environmental Medicine, Aarhus University Hospital, Aarhus, Denmark; 4 Department of Society and Politics, Aalborg University, Kroghstræde 7, 9220 Aalborg Eest, Denmark

**Keywords:** Multidimensional index, Employment precariousness, Stress, General health

## Abstract

**Objective:**

Precarious employment (PE), characterized by job insecurity, instability, low wages, and limited access to social protections, has been linked to adverse health outcomes. This study examines whether PE, measured using a newly validated precarious employment index (PEI), is associated with high stress and poor self-rated health (SRH) in Danish workers.

**Methods:**

We conducted a cross-sectional study including 3339 Danish workers aged 18–45 years. The PEI captures six dimensions of working conditions: instability, salary, power and rights, work task planning, work time planning, and workers’ collective. PEI scores ranged from 0 (no PE) to 100 (most PE) and were dichotomized at the mean + 1 Standard Deviation (SD) into “no PE” and “PE”; 15% were classified as PE. Health outcomes comprised dichotomized measures of Perceived Stress and SRH. Logistic regression models were applied, adjusting for sex, age, ethnicity, highest education, cohabitation, previous mental illness, and labor market participation.

**Results:**

Workers aged 18–24 years had the highest average PEI score (37.7, SD = 12.6). Participants in the PE category had significantly higher odds of reporting high stress (adjusted OR (AOR) = 4.0, 95% CI 3.2‒5.0) and poor SRH (AOR = 3.0, 95% CI 2.3‒3.8). Age-stratified analyses indicated stronger associations among workers aged 30–45 years compared to workers aged 18‒29 years.

**Conclusions:**

Workers aged 18–24 years and those with lower educational attainment had the highest PEI scores. PE was associated with higher odds of high stress and poor SRH, with the strongest associations observed among workers aged 30–45 years.

## Introduction

Precarious employment (PE) is characterized by job insecurity, instability, low wages, and limited access to social protections. As traditional full-time, stable jobs give way to more flexible and often unstable work arrangements, a growing number of workers face increased job-related uncertainty (Benach et al. 2014; Bodin et al. 2020). Employment stability is a well-established determinant of health; thus, the increase in PE raises concerns about potential negative effects on physical and mental well-being (Van Aerden et al. 2015). This shift towards more precarious work arrangements has disproportionately affected women, migrants, and younger workers, who are overrepresented in precarious positions (Bodin et al. 2020). Among these groups, younger workers at the beginning of their careers may be especially vulnerable to the stressors associated with PE (Benach et al. 2014; Kalleberg and Vallas 2017). While PE is often linked to temporary or non-contractual positions, researchers such as Lewchuk and Juliá et al. argue that it also impacts workers in a standard employment relationship (SER) (Juliá et al. 2017; Lewchuk 2017). This perspective emphasizes the necessity for broadening of PE concept beyond contract types, encompassing elements such as unstable work hours, insufficient workplace support, and limited job control (Macmillan and Shanahan 2021; Rodgers and Rodgers 1989; Tompa et al. 2007). Regardless of contractual status, these work conditions contribute to precariousness and its associated health risks.

Extensive research links PE to adverse health-related outcomes. For instance, PE increases the risk of poor mental health outcomes, including psychological distress among younger workers and with effects that intensify with prolonged exposure (Jaydarifard et al. 2023; Pulford et al. 2022; Ronnblad et al. 2019). Similarly, Oddo et al. reports an association between PE and heightened levels of perceived and biological stress (Oddo et al. 2024). Self-rated health, a subjective measure of overall general health, is also negatively impacted by prolonged PE exposure (An and Park 2022; Jaydarifard et al. 2023). These adverse health outcomes may be explained by disparities in employment and working conditions—such as job insecurity, inadequate income, limited workplace rights, insufficient workplace support, and limited job control—that differentiate precarious from more secure forms of employment (Jaydarifard et al. 2023).

While the adverse effects of PE are well documented, inconsistencies in how PE is conceptualized and measured continue to limit comparability across studies. Rönnblad et al.’s systematic review highlighted the detrimental impact of job insecurity on mental health but emphasized the absence of a clear, multidimensional definition of PE (Ronnblad et al. 2019). Kreshpaj et al. similarly called for standardized tools to assess PE across studies, particularly given its high prevalence among younger workers (Kreshpaj et al. 2020). In this context we argue for national adaptations which allow for the addition of potentially context specific aspects in addition to the extensions of the PE discussed above. Multidimensional measurement instruments such as the Employment Precariousness Scale (EPRES) (Vives et al. 2010) and its national adaptations, including EPRES-BE (Vandevenne et al. 2022; Vanroelen et al. 2024) have advanced the measurement of PE across countries. Cross-national research has shown that measurement properties and the relative importance of dimensions may vary across national institutional contexts (Jaydarifard et al. 2023). This suggests that direct transfer of instruments across labor-market regimes may not fully capture context-specific manifestations of PE.

### Precarious employment in the Danish labor market

Understanding PE’s effects requires examining how it functions within specific labor market contexts. The Danish labor market combines liberal employment policies with a relatively strong social safety net, including unemployment benefits and support for job retraining (Bredgaard et al. 2006; Bredgaard and Madsen 2018). These institutional supports may help buffer some of the risks typically associated with PE. Nonetheless, they do not eliminate them entirely. For instance, research by Nielsen et al. shows that even within this relatively supportive labor market, part-time and marginal workers face greater job insecurity and poorer health outcomes than their full-time counterparts (Nielsen et al. 2021a, b).

However, as extensive research has clearly pointed out, we have to put emphasis on the importance of control over working conditions when examining PE’s health impacts (Macmillan and Shanahan 2021; Rodgers and Rodgers 1989; Tompa et al. 2007). This calls for not only focusing solely on contract type or hours worked as this fails to capture the nuanced dynamics of precarious work in this context.

To address these gaps, this study examines whether PE, measured using a newly validated PE Index (Poulsen et al. 2025), is associated with high stress and poor self-rated health among Danish workers. By analyzing these associations within a Scandinavian Welfare regime with a labor market characterized by flexicurity, the study contributes Nordic evidence and advances understanding of how PE manifests beyond contract type in this particular context. To our knowledge, this is among the first studies to apply a nationally developed multidimensional PE measure in such a context to examine its associations with stress and self-rated health.

## Methods

### Study design and population

**Fig. 1:**
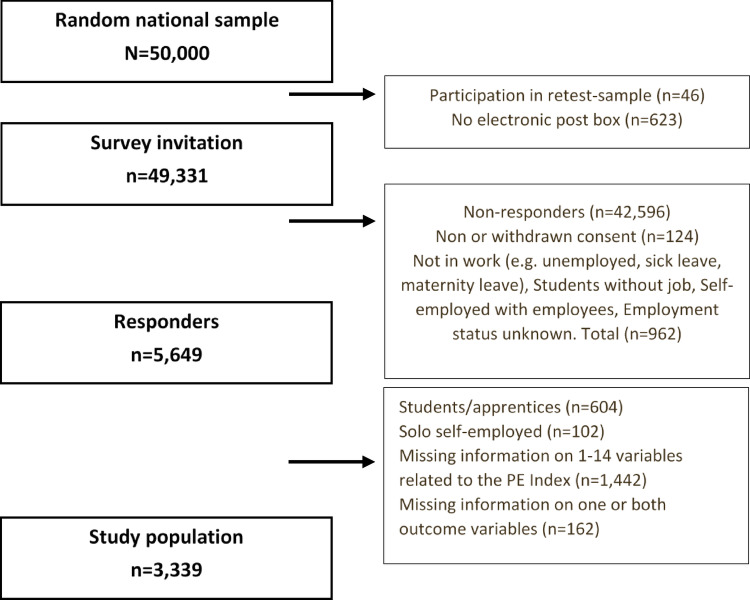
Detailed flowchart for data collection

This is a Danish cross-sectional study using survey data, combined with register-based data in a random sample of employees aged 18–45 years. Invitations to participate in the research project were sent out on November 9, 2023, and access to the questionnaire remained open until the end of March 2024. These data formed the basis for the development of the PE Index (PEI). Details on the data collection process and the development and validation of the PEI are provided in a previous study (Poulsen et al. 2025). Exclusion criteria for this study were as follows: (1) being solo self-employed, given the study’s focus on the worker-employer relationship, (2) being students or apprentices, as the study targeted individuals not currently enrolled in education, (3) having missing data on at least one of the outcomes. Following exclusions, the final study population comprised 3,339 participants. The response rate for the survey was 15.3%. A detailed flow chart can be seen in Fig. 1.

### Definition of outcome

The outcome measures were the Perceived Stress Scale and Self-rated Health; both obtained through the survey.

Perceived stress was assessed using Cohen’s Perceived Stress Scale (PSS) in its 10-item version (Cohen et al. 1983). The PSS-10 has previously been validated in a Danish context (Eskildsen et al. 2015). Responses were given on a 5-point scale ranging from 0 (“never”) to 4 (“very often”), resulting in a total score between 0 and 40. The scale was dichotomized into “low stress” (scores < 18) and “high stress” (scores ≥ 18), based on a cut-off previously used for younger healthy individuals in the Danish National Health Profile (Jensen et al. 2018).

Self-rated health (SRH), a well-established predictor of various health outcomes, served as a key indicator for assessing the impact of PE (Jaydarifard et al. 2023).

SRH was measured using a single item from the SF-36: “In general, would you say your health is…” with the response options *excellent*, *very good*, *good*, *fair* or *poor *(Framework 1992). The variable was coded from 0 (“poor”) to 4 (“excellent”) and subsequently dichotomized into “poor SRH” (poor or fair) and “good SRH” (good, very good, or excellent).

### Definition of exposure: precarious employment index (PEI)

The PEI is a formative index based on six dimensions of employment and individual working conditions: instability, salary, power and rights, work task planning, work time planning, and workers’ collective. The index has previously been developed and validated in a Danish working population, demonstrating acceptable reliability and associations with related constructs including subjective job insecurity, worker vulnerability, and the WHO-5 Well-being Index (Poulsen et al. 2025). The PEI was developed to contribute to the discussion on what may constitute elements of PE for younger workers in Denmark (Nielsen et al. 2019). The index was developed drawing on Bodin et al. (2020) multidimensional framework and adapted to the Danish labor market context.

The six dimensions capture contractual and job continuity (instability), income adequacy (salary), access to, and ability to exercise, work-related rights (power and rights), autonomy in task planning, predictability and control over working hours, and workplace collective support. While these dimensions overlap with core elements of EPRES (Vives et al. 2010) and EPRES-BE (Vandevenne et al. 2022; Vanroelen et al. 2024) (e.g., wages, rights, and working time), the PEI additionally incorporates workplace collective aspects of employment, reflecting the importance of collective support mechanisms in the Danish labor market context (Hasle 2013). The PEI is a sum score of the six dimensions, resulting in a score from 0 to 100, where 0 represents “least PE” and 100 represents “most PE”. No missing information was allowed for any of the items in the index. For analytical purposes, the PE Index score was dichotomized into *no PE* and *PE* using the mean + 1 standard deviation (SD) (Mean = 24.9, SD = 12.0) as the cut-off. It is important to note that this cut-off is specific to the sample population studied.

### Covariates

In Denmark, we can combine data from different administrative registers using a unique personal identification number (CPR number) given to newborn individuals or upon entry to Denmark for immigrants (Schmidt et al. 2014). Included covariates derived from national registers in Statistics Denmark (Statistics Denmark 2024) and the DREAM register (Danish Register for Evaluation of Marginalization) (Hjollund et al. 2007) is described below. With a few exceptions the data derived from these registers is highly reliable. Labor market status and information on educational attainment for immigrant workers are the exception as labor market status is only recorded from when the immigrant has obtained a CPR-number, and education taken abroad is less accurately recorded (Sundhedsdatastyrelsen 2020).

Age and sex were obtained from the CPR register (Schmidt et al. 2014). Highest education was derived from education registers (Jensen and Rasmussen 2011) in Statistics Denmark and categorized into *primary school/upper secondary education* (the two categories were collapsed due to very few in the primary school category)(ISCED 2 & 3), *vocational educational training* (ISCED 3), *short-cycle higher education* (ISCED 5), *medium-cycle higher education* (ISCED 6), *master´s programs or PhD* (ISCED 7 & 8), based on the Danish version of the International Standard Classification of Education (ISCED) (CIRCABC 2022).

Information on cohabitation status was obtained from BEF (Danish Population Register) and dichotomized into *not living alone* or *living alone*. Whether a person lives alone or with a partner may have an influence on the effects of PE (Lewchuk and Laflèche 2017), and may also be associated with the risk of poor mental health (Tamminen et al. 2019).

Ethnicity (*Danish* or *other*) were based on the Statistics Denmark IEPE register (Immigrants and their descendants), which defines country of origin based on documented citizenship if any else self-reported origin. Studies have shown that immigrants are more precariously employed compared to non-immigrants, which may be associated with an increased risk of poorer mental health outcomes, including stress (Koseoglu Ornek et al. 2022).

Poor mental health, including symptoms of stress, may be associated with an increased risk of temporary employment (previously applied as a proxy for PE) (Dawson et al. 2015). Previous mental illness (PMI) was obtained from the Danish Psychiatric Patient Register (Mors et al. 2011) and defined as any psychiatric diagnoses (F00-F99) according to the International Classification of Diseases (ICD) 10th revision for the last five years and dichotomized into (*yes* or *no*).

Labor market participation (LMP) (previous 5 years) was obtained from the DREAM register. DREAM codes were grouped inspired by the work done by Kyndi et al. (2023) and calculated as a mean percentage of weeks in work each of the previous five years. This was then dichotomized into stable attachment (working > 40 weeks per year) and unstable attachment (working ≤ 40 weeks per year), which has been applied in other studies (Biering et al. 2013; Jakobsen et al. 2018). The DREAM register provides weekly information on social benefits and includes information on public transfer payments related to i.e. unemployment benefits, sickness absence compensation, disability pension and state educational grants (Lund et al. 2013). The LMP score was estimated as the cumulative number of weeks in employment, education, or parental leave divided by the total number of weeks potentially eligible for work. The information is included as previous unstable attachment to the labor market may be associated with later PE (Filomena and Picchio 2022).

### Statistical analyses

Descriptive proportions of the study population are presented with number (n) and percentages (%), where the exposure and the outcome variables are presented both in a continuous (Mean (SD)) and dichotomous (n (%)) manner. Furthermore, we present descriptive statistics of the PEI across sex, age, highest education, labor market participation (last 5 years) (LMP), ethnicity, cohabitation status and previous mental illness (PMI) (last 5 years) with n (%), or mean, and SD. We also employed the two-sample Wilcoxon rank-sum (Mann–Whitney) test to examine the distribution of the PEI between groups (sex, cohabitation, ethnicity, PMI and LMP) and applied the Kruskal–Wallis test to assess significant differences in the distribution of PEI across more than two strata (age-groups, highest education).

Multivariate logistic regression models were used to analyze the associations between the dichotomized PEI score and perceived stress, and SRH. Adjustments were made for sex, age, ethnicity, highest education, cohabitation, PMI and previous LMP, an approach applied in earlier studies (Jaydarifard et al. 2023). Estimates for the regression analyses are presented both for the crude and the adjusted analyses. Supplementary regression analyses were performed to investigate potential age differences (18–29 versus 30–45 years) in associations between PE and the two outcomes, as interaction analyses revealed distinct patterns across these measures.

Stata version 18.0 (Copyright 1985–2023 StataCorp LLC, StataCorp, Texas, USA) was applied as the statistical software package.

## Results

### Description of the study population

The study included 3,339 participants, with a higher proportion of women (59%) than men. The average age was 35.3 years (SD = 7.1). Almost 60% were 35–45 years old and less than 10 percent were 18–24 years old. The length of highest education varied, with more than 50% having either medium-cycle higher education or master´s programs/PhD, and around 11% having primary school/upper secondary education. Regarding labor market participation, over the past five years, about 93% had stable labor market attachment. Additionally, about 9% of participants had ethnicity other than Danish, 72% of participants lived with a partner, and finally, about 10% had a history of mental illness within the previous five years.

The mean PEI score was 24.9 (SD = 12.0), with 15% classified as being in PE according to the defined cutoff (PEI ≥ 36.9). On measures of perceived stress, and SRH, mean scores were 15.6 (SD = 6.6), and 2.5 (SD = 0.9), respectively. Notably, 38% of participants were categorized with high stress, and 14% with poor SRH (Table 1).

**Table 1:** Description of the study population, N = 3,339

		Total
		n (%)
Sex	Women	1,956 (59)
	Men	1,383 (41)
Mean age (SD)		35.3 (7.1)
Age-groups	18–24	306 (9)
	25–29	442 (13)
	30–34	660 (20)
	35–39	792 (24)
	40–45	1,139 (34)
Highest education	Primary school/upper sec school	356 (11)
	Vocational educational training	891 (27)
	Short-cycle higher education	143 ( 4)
	Medium-cycle higher education	801 (24)
	Master’s programs or PhD	984 (29)
	Missing	164 (5)
Labor market participation^a^	Stable	3,098 (93)
	Unstable	241 (7)
Ethnicity	Danish	3,016 (90)
	Other	293 (9)
	Missing	30 (1)
Cohabitation	Not living alone	2,393 (72)
	Living alone	916 (27)
	Missing	30 (1)
Previous mental illness	No	3,010 (90)
	Yes	329 (10)
PE index, mean (SD)		24.9 (12.0)
PE index, dichotomous	No PE	2,825 (85)
	PE	514 (15)
Perceived stress, mean (SD)		15.6 (6.6)
Self-rated health, mean (SD)		2.5 (0.9)
Perceived stress	Low stress	2,074 (62)
	High stress	1,265 (38)
Self-rated health	Good	2,880 (86)
	Poor	459 (14)

Labor market participation^a^ was calculated as the mean percentage of weeks in work during the previous 5 years and dichotomized into stable (> 40 weeks/year) and unstable (≤ 40 weeks/year)

Women had higher average PEI scores than men, and younger workers, particularly those aged 18–24 years, had the highest PEI score‒more than 10 points higher than the next age group (25–29 years). Education similarly differentiates PEI scores. Those with primary school/upper secondary education had the highest PEI score, which was approximately 10 points higher than those with vocational educational training and 13.7 points higher than those with master’s programs/PhD, respectively. In addition, singles had higher PEI scores than individuals cohabitating with a partner. Participants with ethnicity other than Danish and those with a history of mental illness or unstable labor market participation consistently had higher PEI scores, compared to their respective counterparts (i.e., Danish participants, individuals without a history of mental illness and those with stable labor market participation) (Table 2).

**Table 2:** Precarious employment index (PEI) in relation to covariates, N = 3339

		PEI (0–100)	
		Mean (SD)	*p*-value
Sex	Women	25.3 (11.9)	
	Men	24.3 (12.0)	0.005^c^
Age-groups	18‒24	37.7 (12.6)	
	25‒29	27.2 (11.0)	
	30‒34	24.2 (10.6)	
	35‒39	23.3 (11.7)	
	40‒45	22.1 (10.7)	< 0.001^d^
Highest education^a^	P/up sec.^b^	35.2 (13.6)	
	VET	25.6 (11.7)	
	SC high	22.6 (10.1)	
	MC high	23.8 (9.8)	
	Master/PhD	21.5 (10.8)	< 0.001^d^
Cohabitation^a^	Living alone	27.9 (12.3)	
	Not living alone	23.8 (11.6)	< 0.001^c^
Ethnicity^a^	Other	27.3 (14.5)	
	Danish	24.7 (11.7)	0.020^c^
Previous mental illness	Yes	27.1 (12.7)	
	No	24.6 (11.9)	0.001^c^
Labor market participation^e^	Unstable	30.6 (13.4)	
	Stable	24.4 (11.7)	< 0.001^c^

Missing (n = 30‒164)^a^

Education abbreviates^b^

*P*/*up sec* Primary school/upper secondary school, *VET* Vocational educational training, *SC high* Short-cycle higher education, *MC high* Medium-cycle higher education, *Master*/*PhD*. Master’s programs or PhD

Wilcoxon rank-sum test^c^

Kruskal–Wallis test^d^

Labor market participation^e^ was calculated as the mean percentage of weeks in work during the previous five years and dichotomized into stable (> 40 weeks/year) and unstable (≤ 40 weeks/year)

### Main results

To understand the potential health impacts of PE, we explored the associations between PEI scores and perceived stress, and SRH.

Perceived stress: In terms of stress, workers in the PE category had increased odds of reporting “high stress” with a crude OR of 4.0 and an adjusted OR (AOR) of 4.0, (95% CI 3.2–5.0) (Table 3). When we carried out age-stratified analyses, the results showed that workers aged 30–45 in the PE category had almost twice the odds of reporting “high stress”, compared to workers aged 18–29, AOR = 5.5 (95% CI 4.0–7.4), AOR = 2.8 (95% CI 2.0–4.1), respectively.

**Table 3:** Crude and adjusted logistic regression analyses for the associations between Precarious employment and Perceived stress or Self-rated health (SRH), N = 3,339

PE index	Number of individuals							
	Perceived stress, all			High stress			High stress	
	High stress	Low stress	n	OR^a^	95% CI^e^	n^c^	AOR^b,d^	95% CI
No PE	926	1899		(Ref)			(Ref)	
PE	339	175		4.0	3.3;4.8		4.0	3.2;5.0
			3339			3175		

PE index	Number of individuals							
	Perceived stress, age 18–29 years^f^			High stress			High stress	
	High stress	Low stress	n	OR	95% CI	n^c^	AOR^d^	95% CI
No PE	165	347		(Ref)			(Ref)	
PE	133	103		2.7	2.0;3.7		2.8	2.0;4.1
			748			736		

PE index	Number of individuals							
	Perceived stress, age 30–45 years^f^			High stress			High stress	
	High stress	Low stress	n	OR	95% CI	n^c^	AOR^d^	95% CI
No PE	761	1552		(Ref)			(Ref)	
PE	206	72		5.8	4.4;7.7		5.5	4.0;7.4
			2591			2439		

PE index	Number of individuals							
	Self-rated health, all			Poor SRH			Poor SRH	
	Poor SRH	Good SRH	n	OR	95% CI	n^c^	AOR^d^	95% CI
No PE	317	2508		(Ref)			(Ref)	
PE	142	372		3.0	2.4;3.8		3.0	2.3;3.8
			3339			3175		

PE index	Number of individuals							
	Self-rated health, age 18–29 years^f^			Poor SRH			Poor SRH	
	Poor SRH	Good SRH	n	OR	95% CI	n^c^	AOR^d^	95% CI
No PE	46	466		(Ref)			(Ref)	
PE	51	185		2.8	1.8;4.3		2.3	1.4;3.7
			748			736		

PE index	Number of individuals							
	Self-rated health, age 30–45 years^f^			Poor SRH			Poor SRH	
	Poor SRH	Good SRH	n	OR	95% CI	n^c^	AOR^d^	95% CI
No PE	271	2042		(Ref)			(Ref)	
PE	91	187		3.7	2.8;4.9		3.3	2.4;4.5
			2591			2439		

Odds Ratio (OR)^a^

Adjusted Odds Ratio (AOR)^b^

Differences in n is due to missing on covariates (highest education, ethnicity and cohabitation)^c^

Adjusted for: sex, age, highest education, ethnicity, cohabitation, previous mental illness, previous labor market participation^d^

95% CI 95% Confidence interval^e^

For the stratified regression analyses, the 18–24 and 25–29 age groups were combined into 18–29 years and the remaining age groups were combined into 30–45 years^f^

SRH: Similarly, workers in the PE category had higher odds of reporting “poor SRH”, with a crude OR of 3.0. After adjusting, the AOR was 3.0 (95% CI 2.3–3.8). In age-stratified analyses, workers aged 30–45 had higher odds of “poor SRH” than those aged 18–29 (AOR = 3.3, 95% CI 2.4–4.5 vs AOR = 2.3, 95% CI 1.4–3.7).

## Discussion

This study demonstrates robust associations between multidimensionally measured precarious employment and both perceived stress and self-rated health in Denmark. Using a Danish PE Index developed and validated in a prior study, we show that PE is associated with health outcomes even within a highly regulated labor market in Scandinavia. The findings therefore contribute Nordic evidence, and support calls for multidimensional measurement in general health research.

In this study population, the proportions of individuals with high stress were slightly higher than in the general Danish population while the proportion with poor SRH was comparable (Berg et al. 2024; Rosendahl et al. 2022). More broadly, an increase in younger people reporting higher stress levels has been observed in recent years (Berg et al. 2024).

The observed associations between PE and the different outcomes are consistent with theoretical pathways linking PE to health through uncertainty of opportunities and reduced control over life situation and planning of the preferred life course (Bodin et al. 2020; Tompa et al. 2007). Notably, the stronger associations among workers aged 30‒45 years may reflect greater financial and caregiving responsibilities and potentially longer cumulative exposure to precarious conditions, suggesting that life-course position can modify the health impacts of PE (Baek et al. 2024).

Our findings indicate that workers aged 18–24 years and individuals with lower education levels experience higher levels of PE, consistent with previous research (Benach et al. 2014). This overlap likely reflects that younger workers often enter the labor market with lower levels of formal education and limited work experience, making them particularly vulnerable to precarious employment conditions.

Even though young workers disproportionally find themselves in jobs with a high level of PE, we find an additional effect of high PE on the different outcomes for the older workers in the sample.

To explore this finding further, longitudinal data are essential, as we need to determine whether entry jobs with a higher level of PE function as steppingstones towards better jobs, or whether there is a lock-in effect that maintains workers in jobs with high PE throughout the working life course. Long-term exposure to poor working conditions, as PE signifies, could account for the stronger effect observed among older workers. In addition, it is important to take into account how this potential scarring effect interacts with different life-course obligations and situations.

### Limitations

The low response rate (15.3%) may limit the representativeness of the study population—particularly among workers aged 18–24 years—and could introduce selection bias. These factors should be considered when interpreting the findings. It is also assumed that individuals in PE are less likely to participate in surveys. A previous study has also experienced challenges in securing participation from workers with alternative employment contracts, which support this claim (Nielsen et al. 2021a, b). Thus, our prevalence estimates are likely conservative, and the observed associations between PE and health outcomes may in fact underestimate the true burden. Furthermore, as public benefits in the DREAM register are generally not available to persons younger than 18 years, the labor market participation scores (last five years) for participants aged 18–22 may overestimate their work participation. This overestimation may also occur because some young individuals are financially supported by their parents without receiving public benefits.

The cross-sectional design limits the ability to establish causality between PE and the observed health outcomes. Although associations between PE and perceived stress, and SRH were found, it is not possible to determine whether PE directly caused these negative health outcomes or whether other factors may contribute. Since data were collected at a single point in time, we cannot determine whether these outcomes existed before entering PE, although analyses were adjusted for previous mental illness (PMI) within the last five years. However, relying on a five-year register window may underestimate previous mental illness, as chronic or well-treated conditions without recent hospital contact are not captured. It is also possible that individuals with higher stress, or poor SRH are more frequently employed in precarious jobs (Dawson et al. 2015). Longitudinal studies would be better suited to clarify causality and the directionality of these relationships.

This study relies heavily on self-reported data for perceived stress, and SRH. Such measures can introduce bias due to participants’ subjective responses, which may be influenced negatively as well as positively by personal perception, mood, or temporary conditions at the time of survey completion (Podsakoff et al. 2003). Consequently, resulting either in over- or under-reporting of health outcomes. However, poor SRH has been shown to predict objective health outcomes and may serve as an early indicator of more severe health issues (Jylhä, 2009). Therefore, the subjective nature of SRH makes it a particularly important metric, as it reflects both physical and mental health perceptions and is highly suitable for the analysis of the article.

Although adjustments were made for several covariates, unmeasured confounders, such as personality traits, coping mechanisms, or additional environmental factors, could have influenced both the likelihood of experiencing PE and the associated health outcomes.

While the multidimensional PEI was used, PE is a complex construct that can vary across studies and contexts. For analytical purposes, we dichotomized our exposure variable (PEI) at a cut-off, defining “PE” as scores above the mean + 1 SD on the continuous scale. This ensures that we can document the effect of PE among the highly exposed within the study population compared to the rest. This decision may limit our ability to fully explore the relationship between PEI and health outcomes across the entire spectrum of PEI scores. However, as we grouped the PEI into only two levels, our results depict a simpler and perhaps more cautious view of the effects than a more complex presentation of the pattern of risk across all PEI scores. To assess the robustness of our findings, we conducted a sensitivity analysis with a stricter cut-off (mean + 2 SD). This confirmed our hypothesis that higher PEI scores are associated with greater odds of high stress, and poor self-rated health, although the narrower sample led to wider confidence intervals (results not shown). Despite this limitation, the consistency of our findings reinforces the conclusion that higher PEI scores are associated with poorer health and higher stress outcomes. Because workers in precarious employment may be underrepresented in our sample and self-reported outcomes may involve some misclassification, our estimates most likely underestimate the magnitude of the true associations. These considerations should be kept in mind when interpreting the findings.

### Interpretation

PE represents an accumulation of disadvantages entailing different dimensions of the employer-employee relationship such as asymmetric power relations. These conditions may act as a chronic stressor by increasing uncertainty and limiting workers’ capacity to plan and exert control over daily life resulting in higher stress and poorer self-rated health.

While perceived stress highlights the potential immediate psychological burden of PE, SRH offers a broader perspective on how individuals perceive their overall health, encompassing both physical and mental health. The association with SRH may reflect broader health-related pathways beyond acute stress, potentially linked to sustained psychosocial strain among workers experiencing precarious conditions.

Future longitudinal research would be necessary to explore how persistent PE affects health trajectories over the course of a worker’s career. Pulford et al. showed that persistent PE is associated with poorer health, particularly when outcomes are assessed over shorter follow up periods (Pulford et al. 2022). In addition, we need to disentangle the possible interlinkage of the effects of life course obligations and scarring effects of prior employment on later employment.

By applying a multidimensional measure of PE within a nationally specific context, this study contributes to international research by illustrating how PE may manifest beyond contract type in a Danish context.

### Generalizability

The study focuses on Danish workers aged 18–45 years, but the impacts of PE may vary depending on age, socioeconomic status, or other factors. Additionally, the study’s findings may be more relevant to countries with labor markets like Denmark’s and may not fully capture the situation in countries with different employment protections or labor laws and regulations.

## Conclusions

Workers aged 18–24 years and those with lower educational attainment had the highest PEI scores. PE was associated with higher odds of high stress and poor self-rated health, with the strongest associations observed among workers aged 30–45 years. Thus, our results showed that workers at the age of 30–45 are disproportionately more affected by PE than workers in their twenties. The cross-sectional design limits causal inference. In addition, self-reported measures may introduce bias, and the low response rate (15.3%) may limit representativeness—particularly among 18–24-year-olds.

Nevertheless, our findings underscore PE as a public health concern even within labor markets with relatively strong welfare protections like Denmark’s. Future research should examine these dynamics longitudinally and across sectors and demographic groups. Efforts to mitigate the health consequences of PE should address both structural labor market conditions and the lived realities of workers navigating insecure employment.

## Data Availability

The datasets for the current study are not publicly available due to participants confidentiality and the datasets being located on a secure server at Statistics Denmark.

## Abbreviations

AOR: Adjusted odds ratio
CPR(-number): Central person registration number
CI: Confidence interval
DREAM: Danish register for evaluation of marginalization
E.g.: Example gratia
ICD: International classification of diseases
ISCED: International standard classification of education
LMP: Labor market participation
OR: Odds ratio
PSS: Perceived stress scale
PE: Precarious employment
PEI: Precarious employment index
PMI: Previous mental illness
SRH: Self-rated health
SF-36: Short form health survey 36
SD: Standard deviation
SER: Standard employment relationship
